# Supplementary material for: Structure-Functional Characteristics of the Svx Protein—The Virulence Factor of the Phytopathogenic Bacterium Pectobacterium atrosepticum
Source: Int J Mol Sci. 2022 Jun 21;23(13):6914. doi: 10.3390/ijms23136914 (PMC9266454; doi:10.3390/ijms23136914)
Supplement: Supplementary file 1 [file ijms-23-06914-s001.zip › Supplimentary figures.pdf]

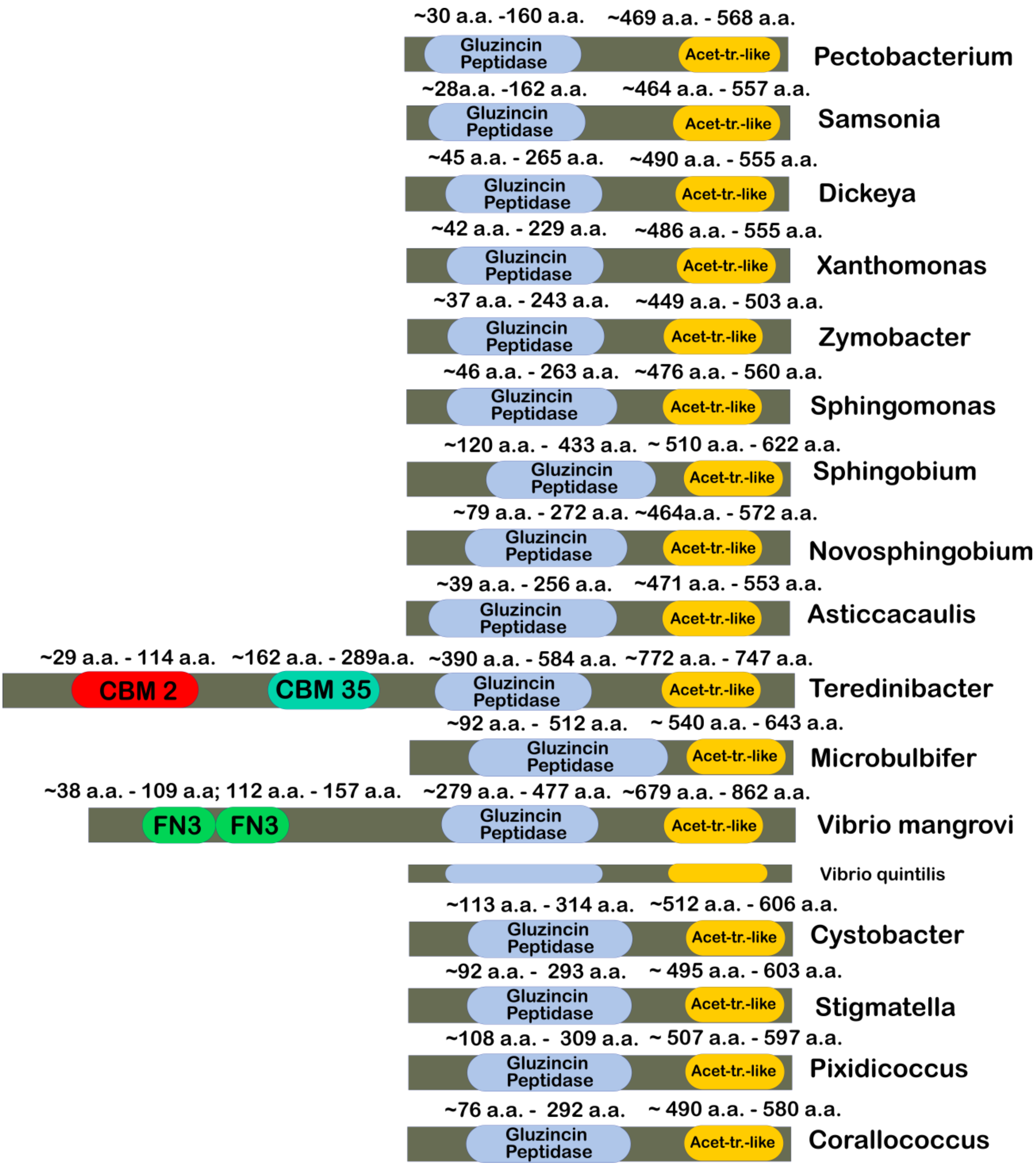

Figure S1. The possible functional domains predicted for Svx-homologues by NCBI Conserved Domain Search, HMMER and Phyre2 servers.

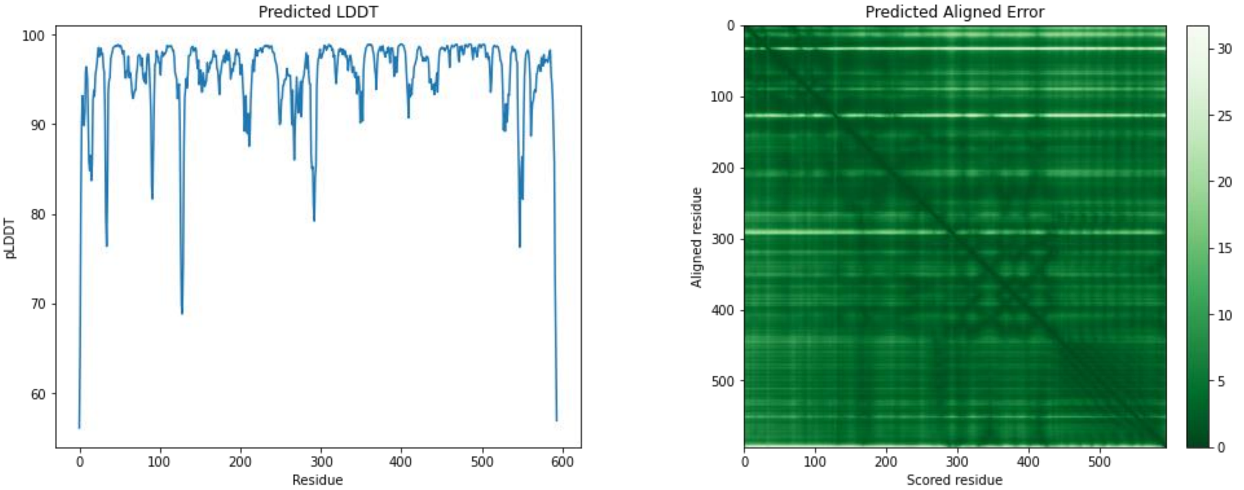

Figure S2. The results of the predicted local-distance difference test for each residue position of the Svx protein of the *Pectobacterium atrosepticum* model built by AlphaFold 2. The model confidence is high, with LDDT above 70% for the majority of the residues. The region of low confidence (slightly below 70%) is located on the loop.

```
WP_011092533.1  X118  -------AGGG--SMGM----------------WIGPASLKDNWGLAHEFTHALQGQ---
ZP_06941093.1   X1274 VNDVAISIGAA--HSGY-PVMNASFNATS----KSLNTAPLNSWLLWHEVGHNAAEA---
WP_001034562.1  X1264 TNDVQISIGDA--HSGY-PVMNSSFSTNS----TTLPTTPLNDWLIWHEVGHNAAET---
NP_001123498.2  X611  VADVQISAGWM--HSGY-PIMCHLESVKEII--NEMDMRSRGVWGPIHELGHNQQRH---
WP_012420550.1  X454  VVDRQISAGAG--HSGY-PAMATKDWTNSIA---TGSIIHSGSWGLWHELGHNHQSP---
ZP_04093101.1   X254  T-SPFMYANNY--LTGY-----AEDSIEFVL---DIEKFTKDGWGPWHEVGHIHQQV---
WP_015834470.1  X204  ----YMFATNG--HMGF-----NGDAALQRL------LTTNNGWGIWHESGHQRQQS---
WP_011590734.1  X723  SGGAFMNAGNG--VIGIRPG-----------NQDAILAANKGWGVAHELGHNFDTG---
WP_008764444.1  X536  --GSYMWASDY--QIGF-----VYTYLGNILLEDNVMAAEDNAWGPAHEIGHVHQAA---
XP_001330197.1  X317  NFDSYVPAGAAVAFVGANFIQAPFSWSTAMI---NYEGAKWGGWGNVHEYGHHFQSG---
XP_654508.1     X343  NFDQRVDAGAAVAYVGRWFTQNPSDWAAACV--GKDGLINYGNWGPLHEMNHHMQGTYLK

WP_011092533.1  --TGGFQGAGGDDYVGWIWESHANWMTHQMDEFR-GTSAHCSEMQVNYSHIYLGST-RNR X205
ZP_06941093.1   --PFNVDGAT---------EVVNNLLALYMQDCHLGKMARVEQ------DIRIAP----- X1361
WP_001034562.1  --PLNVPGAT---------EVANNVLALYMQDRYLGKMNRVAD------DITVAP----- X1351
NP_001123498.2  --GWEFPPHT--------TEATCNLWSVYVHETV-LGIPRAQA------HEALSP--PER X703
WP_012420550.1  --PFTMEGQT---------EVSVNIFSMVCEVMG-TGKDFESCW-----GGGMGP--YGM X545
ZP_04093101.1   --PWLSEGMG---------ETTVNIYSLAVQLAF-GNKSRME-------------V-DGR X333
WP_015834470.1  --PYTWSGGTG------MTEVTVNLYSLAVQEGF---HDRASFI-------------DKY X279
WP_011590734.1  -------GRT-------IVEVTNNMMPLFFESKY-KTKTRIT-------DQNIWE--NNT X801
WP_008764444.1  ---INWASST---------ESSNNLFSNFIIYKLGKYKSR---------GNGLGSVATAR X622
XP_001330197.1  ---WGISGTG---------ETTNNVINFITYAML-TEIDATR-------QITLGGA-SFN X409
XP_654508.1     GGNWGISNPG---------EETNNVMTSINYILY-TNIAGHR-------NQGLSG----- X438
```

Figure S3. The alignment of the amino acid sequence of the Svx protein of *Pectobacterium atrosepticum* (*Pba*) (NCBI ID WP_011092533.1) with M60 family metallopeptidases of *Vibrio cholerae*: (NCBI ID ZP_06941093.1), *Escherichia coli* (NCBI ID WP_001034562.1); *Bacillus thuringiensis* (NCBI ID ZP_04093101.1), *Photorhabdus asymbiotica* (NCBI ID WP_015834470.1), *Bacteroides thetaiotaomicron* (NCBI ID WP_008764444.1), *Trichomonas vaginalis* (NCBI ID XP_001330197.1), *Entamoeba histolytica* (NCBI ID XP_654508.1), *Homo sapiens* (NCBI ID NP_001123498.2), *Akkermansia muciniphila* (NCBI ID WP_012420550.1), *Clostridium perfringens* (NCBI ID WP_011590734.1) (Nakjang et al., 2012). The conservative zinc-binding motif HEXXHX(8,28)E is colored in red and carbohydrate binding residues are coloured in green. The alignment was built using the MAFFT algorithm and the BLOSUM62 substitution matrix.
